# The Use of Novel Alginate Capsules in a Monitoring System for *Drosophila suzukii* in a Cherry Orchard in the Region of La Araucanía, Chile

**DOI:** 10.3390/insects16010013

**Published:** 2024-12-27

**Authors:** Marcelo Lizama, Fernando Manuel Alves-Santos, Luis Manuel Navas-Gracia, Daniel Martínez-Cisterna, Cristian Medina, Ramón Rebolledo, Manuel Chacón-Fuentes, Leonardo Bardehle

**Affiliations:** 1 Program of Doctorado en Ciencias Agroalimentarias y Medioambiente, Facultad de Ciencias Agropecuarias y Medioambiente, Universidad de La Frontera, Av. Francisco Salazar 01145, Casilla 54-D, Temuco 4811230, Chile; m.lizama04@ufromail.cl; 2 Program of Doctorado en Ciencias e Ingeniería Agroalimentarias y de Biosistemas, University of Valladolid, 34004 Palencia, Spain; 3 Applied Entomology Laboratory, Facultad de Ciencias Agropecuarias y Medioambiente, Universidad de La Frontera, Temuco 4811230, Chile; d.martinez11@ufromail.cl (D.M.-C.); ramon.rebolledo@ufrontera.cl (R.R.); 4 Departament of Vegetal Production and Forest Resources, University of Valladolid, 34004 Palencia, Spain; fmalsa@uva.es; 5 Sustainable Forest Management Research Institute, University of Valladolid-La Yutera Campus, 34004 Palencia, Spain; 6 TADRUS Research Group, Departament of Agricultural and Forestry Engineering, University of Valladolid, 34004 Palencia, Spain; luismanuel.navas@uva.es; 7 Program of Doctorado en Ciencias de Recursos Naturales, Universidad de La Frontera, Temuco 4811230, Chile; 8 Department of Physics and Chemistry, Facultad de Ingeniería, Universidad Autónoma de Chile, Av. Pedro de Valdivia 425, Providencia 7500000, Chile; cristian.medina@uautonoma.cl; 9 Agriaquaculture Nutritional Genomic Center, CGNA, Temuco 4781158, Chile; manuel.chacon@cgna.cl; 10 Departamento de Producción Agropecuaria, Facultad de Ciencias Agropecuarias y Medioambiente, Universidad de La Frontera, Temuco 4811230, Chile

**Keywords:** pest, microencapsulated, attraction, optimization, fruit trees

## Abstract

Spotted wing drosophila (SWD) is a pest that causes damage due to the female laying eggs under the skin of ripe fruit, from which a larva emerges, causing its collapse and reducing its commercial value. Due to the importance of this pest, monitoring its population is the starting point for any control program; however, there is no early monitoring plan within management tasks, nor are there studies on behavior, the optimization of traps, or their baits. This research proposes the evaluation of a monitoring system with encapsulated baits and adhesive traps that allow effective control. The encapsulated bait was selected after evaluating three options in olfactometric tests in the laboratory; the most attractive bait was WVM, with 70% of visits to the stimulus and 30% to its control, unlike SAG I and SAG II, whose values did not exceed 40% attraction. Among the expected results is the availability of a new format of attractive bait for SWD with a better release rate over time, and the information obtained will allow the generation of SWD population curves for the area, which is essential for decision-making. This study will contribute from the perspective of nanomaterials, insect biology, agricultural entomology, and pest monitoring.

## Simple Summary

This study addresses the pest spotted wing drosophila (SWD), known for causing damage by laying eggs under the skin of ripe fruit, leading to fruit collapse and loss of commercial value. Monitoring SWD populations is critical for control efforts, but current management lacks early detection systems and the optimization of traps and baits. This research evaluates a monitoring system using encapsulated baits and adhesive traps for effective pest control. Laboratory olfactometric tests identified WVM bait as the most attractive, with 70% of visits compared to 30% for the control, outperforming SAG I and SAG II, which showed less than 40% attraction. The study aims to provide a new bait format for SWD with improved release rates over time and generate population curves for the area, essential for decision-making. The research contributes to advancements in nanomaterials, insect biology, agricultural entomology, and pest monitoring.

## 1. Introduction

*Drosophila suzukii* (Matsumura) (Diptera: Drosophilidae), commonly known as the spotted wing drosophila (SWD), is a dipteran insect belonging to the family Drosophilidae. This species was first reported in Japan, causing damage to cherries in 1916, but it was not formally described by Matsumura until 1931, when it was named SWD or cherry fruit fly [1,2]. The first record of this dipteran outside Asia was in 1980, specifically in Oahu, Hawaii (USA) [3]. By 2008, its presence was recorded in North America and Europe. In North America, it was reported in the state of California, and by 2010, it was invading different states such as Utah, Louisiana, North Carolina, South Carolina, Wisconsin, and Michigan [4] before reaching British Columbia (Canada). Subsequently, in 2011, it was detected in the state of Michoacán, Mexico [5]. In Europe, the first records of SWD were in Spain (Cataluña) and Italy (Toscana), expanding in 2012 to the Iberian Peninsula [3]. Only one year later, the first records of SWD in South America occurred. In 2013, the presence of SWD was reported in the Brazilian states of Santa Catarina and Rio Grande [6]. Later in the same year, it was detected in Uruguay (Montevideo and in the rural area of Empalme Maldonado) [7]. For Argentina, the presence of SWD was recorded in 2014 in blueberries, in the town of Lobos, province of Buenos Aires [8]. Bizama et al. [9] report interesting background information on the presence of SWD in Chile. The first record of this occurrence was in 2016; however, this identification was erroneous, which was corrected in time by EPPO [10], confirming its absence in the country for that year. However, in 2017, the Agricultural and Livestock Service (SAG), through the specific surveillance system for the pest, detected SWD for the first time in Chile through the capture of adult specimens in traps installed at the border crossing with Argentina, Mamuil Malal (Villarrica–Pucón path, 39°34′43″ S, 71°29′16″ W) in the La Araucanía Region, southern Chile [11].

The damage caused by this species is attributed to the oviposition of eggs beneath the skin of ripe or ripening fruits. For example, females lay 1–3 eggs per fruit in up to 7–16 fruits per day [12]. Larvae hatch and feed on the fruit pulp, resulting in a loss of turgor, which leads to fruit collapse and a reduction in its commercial value [13]. In the USA, SWD caused annual losses estimated at USD 511 million in 2008, affecting crops such as strawberries, cherries, raspberries, blueberries, and blackberries [14]. In Maine (USA), studies have projected that the economic impact of SWD on wild blueberries could amount to approximately USD 6.8 million, with a 30% reduction in production [15].

In Chile, predictive models have been developed to estimate the potential impacts of SWD on grapevines and cherry trees, indicating that infestation levels could reach 56% and 79%, respectively [9]. Before implementing any pest management plan, it is crucial to understand the behavior and life cycle of the pest throughout the season [16]. Integrated Pest Management (IPM) programs for SWD commonly use commercially available traps and lures for monitoring [17]. Various attractants, such as apple cider vinegar, wine, yeasts, and their mixtures, have been evaluated to identify the most effective components for characterizing SWD pest pressure, yielding mixed results [18,19,20,21,22,23]. Another widely used method is the use of liquid baits and mass trapping. However, both methods have some drawbacks due to the need for timely bait replacement, difficulty in SWD identification due to the need for sifting, and the presence of microorganisms (fungi) [24,25,26,27].

Thus, the search for new alternatives becomes essential to support the work carried out by the SAG, the state agency responsible for environmentally friendly quarantine surveillance, and to apply new technologies. The FAO/WHO emphasizes the importance of new studies and applications, particularly those exploring the interaction of nanomaterials with biological systems, dose–response relationships, and life cycle attractant products. These innovations could improve efficiency by preventing the rapid degradation of substances or mitigating low capture rates due to problems in the release rate of attractants [28,29,30].

Based on the above, encapsulation offers a promising approach for developing new formulations and systems capable of enhancing the efficiency of such products [30]. Encapsulation systems are used for the controlled release of agents for pest control and the delivery of nutrients to plants, making them a valuable tool for the application of bioactive natural products to control economically significant pests [31]. This technique involves depositing a coating material in liquid form onto the material to be encapsulated, which is dispersed as small particles or droplets [32,33]. Encapsulation technology has numerous applications in fields such as drug development, cosmetics, food technology, phytosanitary products, and agriculture [34]. Therefore, this study aims to determine the effectiveness of encapsulated bait within an alginate matrix, in the monitoring and capture of SWD in a cherry orchard in the La Araucanía region, southern Chile.

## 2. Materials and Methods

### 2.1. Insect Collection

Pupae of SWD were obtained from Biofuturo Ltd. (Fundo Alianza Km 11, Camino Traiguén—Galvarino, La Araucanía Region, Chile) and transported to the Laboratory of Applied Entomology at the Faculty of Agricultural and Environmental Sciences, University of La Frontera (Temuco, Chile). The pupae were placed in cubic acrylic containers (45 × 45 × 45 cm) on moistened absorbent paper to prevent dehydration. They were kept at a temperature of 22 ± 5 °C, with 65 ± 5% relative humidity and a photoperiod of 14:10 h (L:D). Upon adult emergence, the insects were captured immediately using a mouth aspirator and individually confined in vials for subsequent bioassays [35]. Only 48 h old virgin females were used in the olfactometric bioassays. Males were discarded because they do not cause damage to the fruit.

### 2.2. Monitoring Traps for SWD

**Figure 1:**
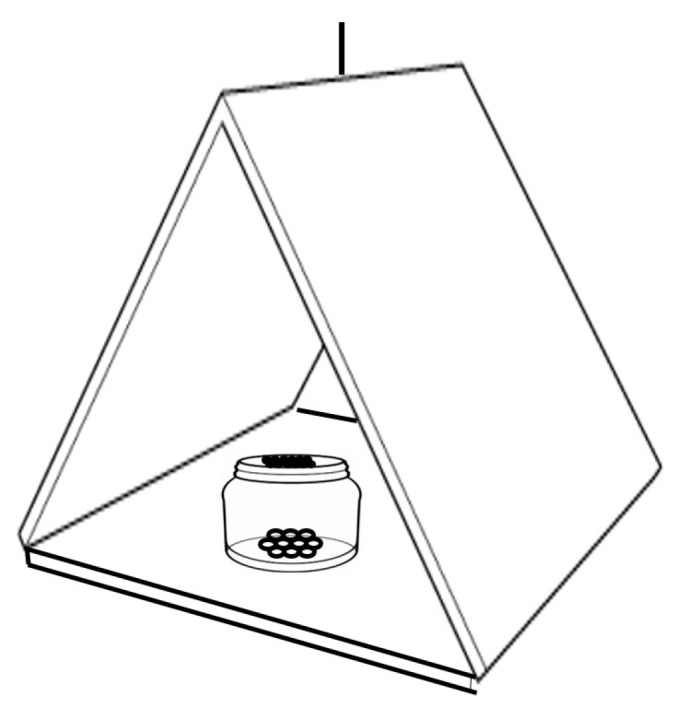
Diagram of the delta-type trap used in this study. The plastic container where the microencapsulated bait and control are deposited is shown in the center of the trap.

A “delta”-type trap exclusively designed for SWD (Sanidad Agrícola ECONEX, S.L., Murcia, Spain) was used in this study. The trap consists of a 0.3 mm thick sheet of red polyethylene terephthalate (PET) coated on its inner surface with an adhesive layer for insect retention. It is also equipped with a hanger and features holes at the corners for hanging when folded (Figure 1). The color red was chosen based on studies by Lee et al. and Renkema et al., which identified red as one of the most attractive colors for SWD [20,36].

### 2.3. Development of Baits for SWD

The baits were prepared following the methodologies outlined in their respective formulations under laboratory conditions (Table 1). SAG I and SAG II were prepared according to the methods proposed by Lasa et al. and Carroll, which are currently utilized by the SAG [37,38,39]. WVM, commonly used for monitoring and capture in Brazil, was also included [40].

### 2.4. Y-Tube Olfactometric Bioassays

The Y-olfactometer has an inner diameter with a central zone 11 cm long and 2 arms 9 cm long. The central zone is connected through a silicone hose to a vacuum pump, a regulator, and a flowmeter to generate a constant flow of 1.8 L min^−1^ in both arms. The arms are connected to glass tubes tapered at their ends, similar to a Pasteur pipette, containing filter paper impregnated with 10 µL of each bait and 10 µL of distilled water as a control. A piece of gauze was used in both arms of the tube to prevent the insect from escaping. The insects were individually introduced through the hole located at the end of the neck of the tube, and the insect’s response was evaluated for a maximum time of 3 min. If the insect made a decision, either control or stimulus (baits), the test was considered successful and terminated at that time. Conversely, insects that showed no response and did not make a decision were eliminated, and the test was considered unsuccessful [41]. After each test, the olfactometer was washed with neutral soap and every five repetitions, the position of the stimulus and control was changed in order to eliminate any factor that could intervene in the preference of the insects. Approximately 120 olfactometries were carried out, of which 90 were successful (30 per bait).

### 2.5. Encapsulation Process Development

The encapsulation of the bait that elicited the strongest attraction response from SWD in the olfactometer bioassays (Section 2.4) was performed following the methodology described by Riyajan et al. [42]. A 100 mL sample of the attractive bait was mixed with 3% sodium alginate (3 g/100 mL), giving the solution a thick and viscous consistency. The sample was then placed on a magnetic stirrer (SCI LOGEX MS-H-PRO, Rocky Hill, CT, USA) at 100 rpm and 40 °C. Once the alginate was fully homogenized, the solution was allowed to cool to room temperature. Using a peristaltic pump (FPP-LabV3, Drifton, Hvidovre, Denmark), the mixture was transferred by gravity into another container, which was continuously stirred with a calcium chloride solution in distilled water (1 g/100 mL), facilitating the formation of microcapsules. The mixture was left to rest for 12 h to ensure the integrity of the microencapsulated bait pearls. The same procedure was followed for the control bait, in which 3 g of sodium alginate was added to 100 mL of distilled water, and the solution was pipetted through the peristaltic pump into the calcium chloride and distilled water mixture.

### 2.6. Release Rate of the Selected Encapsulation

After selecting the baits based on the parameters outlined in the previous section, the WVM treatment demonstrated the best response and was subsequently chosen to evaluate the release rate within an alginate matrix. For this evaluation, 12 g of the bait was weighed and placed in an environmental chamber (Biobase^®^, model BJPX-A250II, Biobase Biodusty, Jinan, China), which controls temperature, light, and humidity. To gather further information on the bait’s behavior and durability over time, references were made to studies by Kanzawa and Hamby [1,43]. The behavior of SWD at its optimal proliferation temperatures (15° to 25 °C) was compared with the average temperatures and relative humidity recorded between September and December 2022 (Table 2), corresponding to the cherry season in the La Araucanía region [44]. Additionally, to determine the release range and encapsulation capacity of the alginate, a solid-phase microextraction (SPME) assay was conducted. Briefly, 12 g of alginate beads designed with the WVM bait was placed in a glass vial. A SPME fiber (DVB/CAR/PDMS, 120 µm × 20 mm) was attached to this vial, allowing it to capture volatile compounds for 2 h. The volatile compounds emitted by these capsules were determined every 24 h, up to a total of 120 h. The major compounds, as well as the total emission of volatiles, were analyzed using a gas chromatograph (GC-2030 Nexis^®^; Shimadzu, Kyoto, Japan) equipped with a mass spectrometer detector. The capillary column used was an Rtx-5MS (30 m × 0.25 mm, 0.25 μm df; Restek GC Columns, Bellefonte, PA, USA). Helium was used as the carrier gas. The column temperature profile was set as follows: 40 °C for 1 min, which was then increased to 280 °C at a rate of 20 °C/min and held at this temperature for 3 min. The injector and interphase were set to 280 °C, and the detector was held at 230 °C. The electron impact ionization energy was set at 75 eV. A 1 μL aliquot from all agri-food byproducts was injected into the GC-MS.

In light of the above, a release rate test was conducted at 21.2 °C and 75% humidity to assess the bait’s weight loss over time. Weight measurements were taken every 2 h during the first 24 h, and then at 48, 72, 96, and 120 h, using an analytical balance (Precisa, model XB 220, Precisa Gravimetrics AG, Dietikon, Switzerland). The collected data were used to perform release kinetics analysis, applying the mathematical models of Korsmeyer–Peppas and Higuchi [45,46].

The release data were processed in triplicate for each time point, allowing for the construction of the release profile of the matrices containing the volatiles for further analysis.

### 2.7. Field Trial with Traps and Encapsulated Bait

The traps were installed at the Maquehue Experimental Field, located 17 km south of Temuco, in the La Araucanía Region, which belongs to the Faculty of Agricultural and Environmental Sciences at the University of La Frontera. The evaluated orchard is divided into three continuous zones, containing Stella, Regina, and Lapins cherry varieties, with planting distances of 4 × 3 m. The trees are approximately 25 years old and are trained in a vase or multi-axis system, which is a common training system in Chile. Drip irrigation is employed, utilizing irrigation tapes and drippers for each unit, and each tree is watered daily. No cherry orchard was subjected to the application of agrochemicals (pesticides or fertilizers) during the season related to the experiment.

The control bait (pure alginate) was alternately placed with the WVM attractive bait across the three cherry varieties mentioned previously. To minimize edge effects potentially caused by adjacent factors, the first planting rows were discarded during installation. The traps were hung on the cherry trees with a wire and with a distance of 4 m between trees. This was repeated in the three varieties of cherry trees mentioned above [47]. Trap density was adjusted following the guidelines of Groupe de travall Baies and SAG, with three rows of six traps each, totaling eighteen units (six per variety). The height of trap placement followed SAG’s recommendations, specifying that traps be placed within the plant/tree foliage, near the presence of fruit, and should not be directly exposed to the sunlight [24,48]. Once the traps were installed, the bait and control were placed. The microencapsulates of each treatment were placed in a 30 mL plastic container in quantities of 12 g/container. Small perforations were made in the lid of each container to allow the dispersion of the bait and control odor. Each container was placed in the central area of the trap to allow individual SWD to enter through any of its entrances (Figure 1).

The installation period began in August 2022, and the traps remained in place until the end of the orchard’s productive season (December 2022). This month was selected because it coincides with the increase in average temperatures to 10 °C, the threshold at which SWD has been reported to initiate its activity [1,43]. Starting at this zero-visit point allowed for the analysis of the insect’s peak population.

The microencapsulated baits were replaced every 25 days, and the traps were inspected weekly. Insects captured in the traps were collected every 7 days and transferred to the Applied Entomology Laboratory at the University of La Frontera for identification using entomological keys [49].

### 2.8. Statistical Analysis

Statistical analyses were performed using Statistix 10 (Tallahassee, FL, USA). To compare the olfactometric responses between the stimulus and the control in female SWD, a generalized linear model (GLM) using the Poisson distribution with a logarithmic link function was performed. To measure the release rate in baits, the Korsmeyer–Peppas model was applied, plotting the logarithmic function of measurement time (hours) on the *x*-axis and the logarithmic function of release percentage on the *y*-axis. For the Higuchi model, the same raw data were processed by converting the number of hours into the percentage of the square root of the corresponding hours for the *x*-axis, and the release percentage was plotted on the *y*-axis [50,51]. Finally, SPME compounds and the field capture data were analyzed using the GLM described above (*p* ≤ 0.05).

## 3. Results

### 3.1. Olfactometric Bioassays

**Figure 2:**
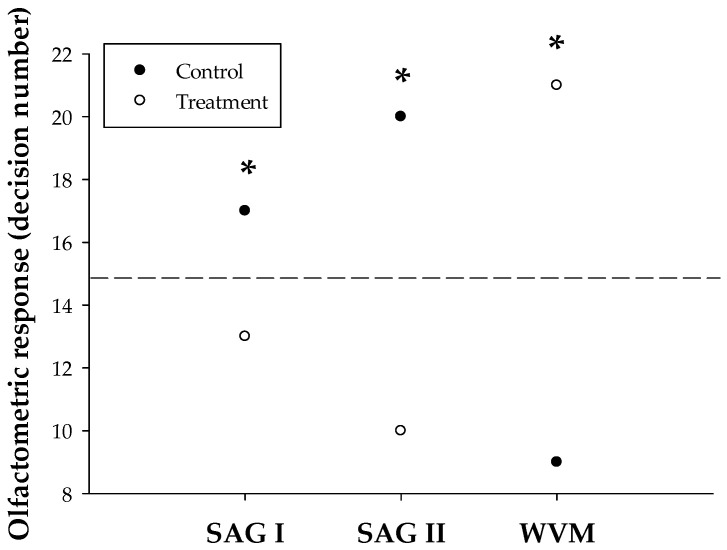
The olfactometric response of female *Drosophila suzukii* to three different attractive baits in a Y-tube olfactometer. SAG I (apple cider vinegar with 95% ethyl alcohol); SAG II (sugar, yeast, wheat flour, apple cider vinegar, and water); and WVM (Merlot wine, apple cider vinegar, and sugarcane molasses). * Indicates a statistically significant difference between treatment and control, according to Poisson distribution.

The results from the olfactometry tests revealed significant differences in SWD preferences (Figure 2). In both SAG I and SAG II treatments, SWD females preferred the control over the stimulus (17 and 20 visits, respectively) (*p* = 0.0005 and *p* = 0.001, respectively). In contrast, with the WVM treatment, females significantly preferred the stimulus (21 visits) compared to the control (9 visits), i.e., there was a preference of 70% versus 30%, respectively (*p* = 0.0097). Based on these results, WVM was selected for encapsulation and further evaluation under field conditions.

### 3.2. Release Rate

**Figure 3:**
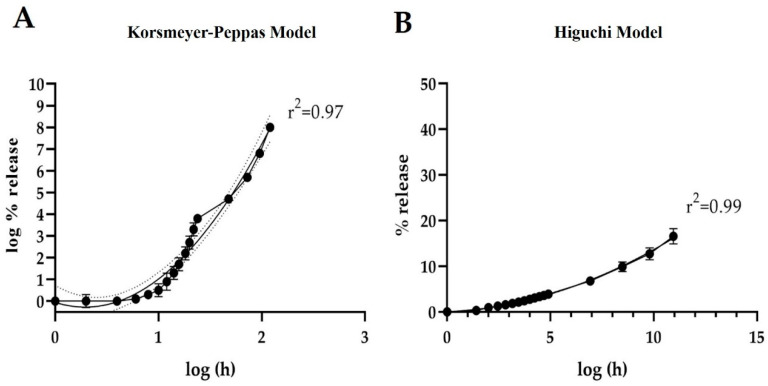
The release rate of the WVM bait in a controlled environment chamber is at the average temperature and humidity for the area (21.2 °C and 75% RH). Mathematical modeling was employed using Korsmeyer–Peppas with the logarithm of time (Log (h)) (**A**) and Higuchi through the square root of time (√ (h)) (**B**).

The selected WVM bait shows a similar behavior, independent of the model, in terms of the release of compounds over time and subject to the average temperature and relative humidity conditions of the area during the cherry season, which runs from September to December (average temperature of 21.2 °C with 75% relative humidity) (Figure 3). Both models, Korsmeyer–Peppas (r^2^ = 0.97) and Higuchi (r^2^ = 0.99), indicated that the release of compounds from the WVM bait reached 20% at 120 h. By projecting the bait’s durability under the conditions mentioned, it is estimated that the complete loss of encapsulated compounds will occur within 25 days.

**Figure 4:**
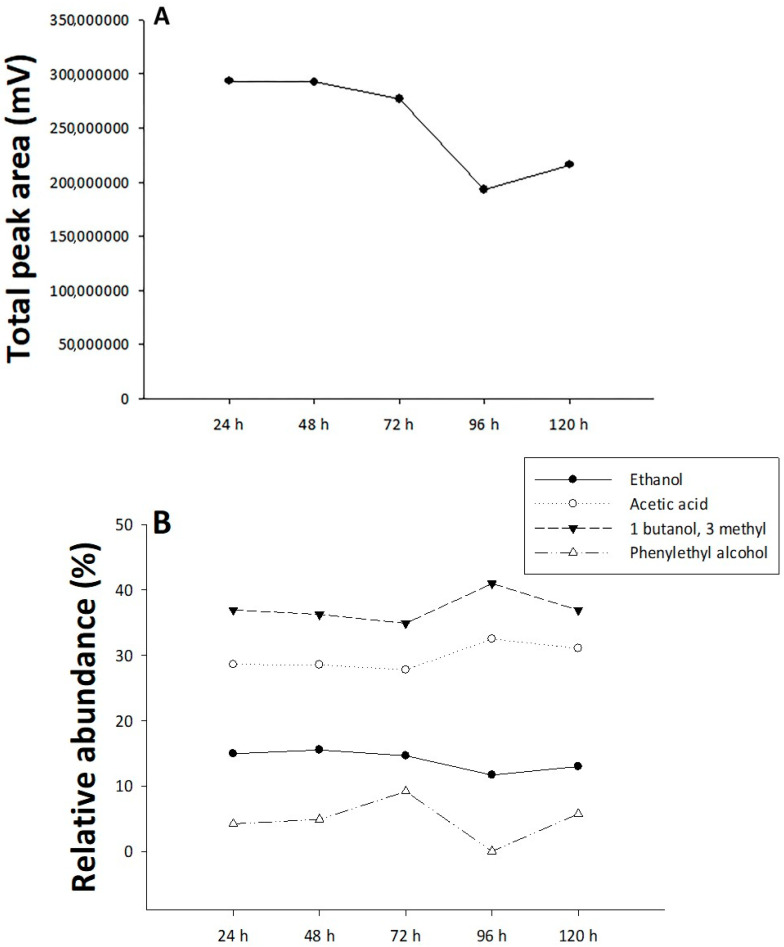
Volatile compound analysis over time. (**A**) Total peak area (mV) for all detected volatiles at different time points (24, 48, 72, 96, and 120 h). (**B**) Relative abundance (%) of individual compounds: ethanol (●), acetic acid (○), 1-butanol, 3-methyl (▲), and phenylethyl alcohol (△). The data points represent the relative abundance of each compound at the specified times. The absence of letters indicates no significant differences in the compounds in relation to time.

Figure 4A shows the following values at different time points: 293,305,351 at 24 h; 292,312,663 at 48 h; 276,440,152 at 72 h; 192,811,652 at 96 h; and 216,009,183 at 120 h. Despite these fluctuations, statistical analysis revealed no significant differences between the groups (*p* = 0.998), indicating that the observed variations were not large enough to be considered statistically significant. This suggests that the passage of time did not have a substantial impact on the measured variable. In addition, Figure 4B shows the relative abundance percentage of each compound at different time points. For ethanol, the abundance fluctuates slightly, with values of 14.93% at 24 h, 15.52% at 48 h, 14.64% at 72 h, 11.68% at 96 h, and 12.99% at 120 h (*p* = 0.0768), indicating no significant difference in its relative abundance over time. Acetic acid, with percentages of 28.60% at 24 h, 28.54% at 48 h, 27.78% at 72 h, 32.51% at 96 h, and 31.06% at 120 h (*p* = 0.121), also shows small fluctuations, and no significant difference is observed. For 1-butanol, the abundance starts at 36.95% at 24 h, decreases slightly to 36.26% at 48 h, and further declines to 34.90% at 72 h before rising to 40.99% at 96 h and then dropping back to 36.92% at 120 h (*p* = 0.342), suggesting no significant change in its relative abundance. Lastly, phenylethyl alcohol shows a more pronounced variation, starting at 4.21% at 24 h, increasing to 4.89% at 48 h, peaking at 9.17% at 72 h, dropping to 0% at 96 h, and rising again to 5.72% at 120 h (*p* = 0.342), indicating no significant difference in its relative abundance across time, despite the clear temporal fluctuations.

### 3.3. Field Trials

**Figure 5:**
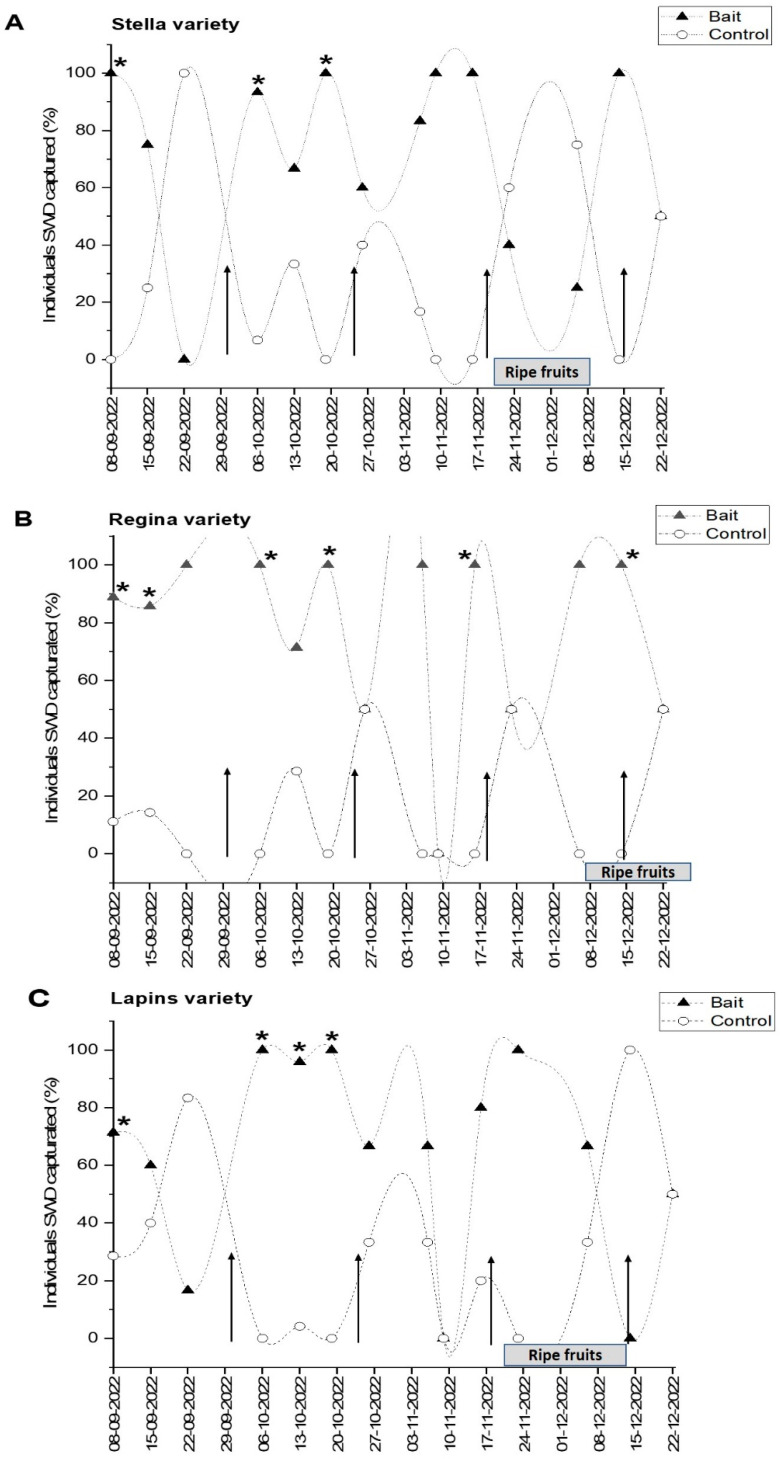
Weekly capture of *Drosophila suzukii* (%) in the period September–December 2022 in a cherry orchard in the La Araucanía region, Chile. (▲) Corresponds to the microencapsulated bait and (○) corresponds to the control. Weekly captures (%) of *D. suzukii* in the Stella variety (**A**). Weekly captures (%) of *D. suzukii* in the Regina variety (**B**). Weekly captures (%) of *D. suzukii* in the Lapins variety (**C**). Black arrows indicate the dates of change of bait and control. * Indicates a significant difference between treatment and control according to the t-student test (*p* ≤ 0.05).

The adhesive traps with the corresponding microencapsulated baits were deployed on 31 August 2022, at the Maquehue Experimental Field, and 14 monitoring visits were conducted throughout the season to track the SWD population. Subsequently, the first record of SWD capture was in the week of 8 September, in traps located in the three varieties of cherry. SWD capture in the Stella variety is shown in Figure 5A. It can be seen that there was generally a higher capture in the traps containing the WVM bait compared to the control. In the weeks of 8 September, 6 October, and 20 October, this difference was significant (100% vs. 0%, *p* = 0.014; 93% vs. 7%, *p* = 0.0007; and 100% vs. 0%, *p* = 0.014, respectively). In the Regina variety, there was a greater number of weeks with significant captures of SWD. In total, there were six weeks where the WVM bait captured significantly more flies than the control: 8 September, 15 September, 6 October, 20 October, 17 November, and 15 December (89% vs. 11%, *p* = 0.019; 86% vs. 14%, *p* = 0.05; 100% vs. 0%, *p* = 0.008; 100% vs. 0%, *p* = 0.0005; 100% vs. 0%, *p* = 0.004; and 100% vs. 0%, *p* = 0.045, respectively) (Figure 5B). In the case of the Lapins variety, significant differences were obtained in four weeks regarding the capture of SWD, in which there was again a higher percentage in the WVM bait traps compared to the control. The weeks with this significance were 8 September, 6 October, 13 October, and 20 October (71% vs. 29%, *p* = 0.023; 100% vs. 0%, *p* = 0.045; 96% vs. 4%, *p* = 0.000004; 100% vs. 0%, *p* = 0.001, respectively) (Figure 5C). Interestingly, when analyzing the weeks with significant captures, most are concentrated in October, after the first bait change (30 September).

**Figure 6:**
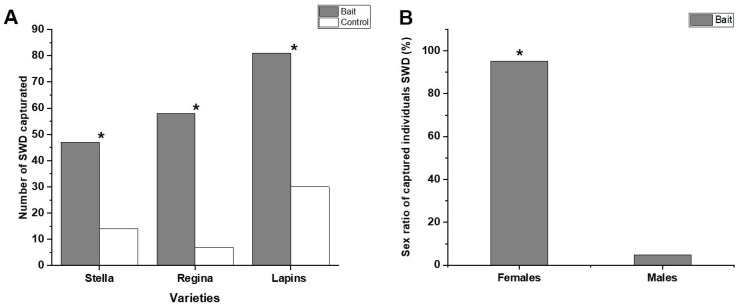
Total number of individual *Drosophila suzukii* captured with the WVM bait in delta-type traps placed in different cherry varieties (Stella, Regina, and Lapins) at the Maquehue Experimental Field, La Araucanía region. * Indicates a significant difference between treatment and control according to Student’s t-test (*p* ≤ 0.05) (**A**). Sex ratio of females and males captured with the WVM bait during the duration of the trial. * Indicates a significant difference between females and males captured, according to the Poisson distribution (*p* ≤ 0.05) (**B**).

When analyzing the total SWD capture in relation to the varieties, it can be observed that Stella (*p* < 0.001), Regina (*p* = 0.008), and Lapins (*p* < 0.001) had a significant effect on the attraction of the dipteran in relation to the control (Figure 6A). However, when analyzing the preference of SWD for any particular variety, no differences were found between them (*p* = 0.6101). Regarding the sexual proportion of SWD, this was significant (*p* < 0.001) because the capture of females in the WVM bait in the three cherry varieties reached 95.3% compared to the control (4.7%) (Figure 6B).

Furthermore, when these results are extrapolated to one hectare (using a planting framework of 4 × 3 m, resulting in a density of 8333 cherry trees/ha), the results show the number of flies per hectare (flies/ha) captured in traps with bait (WVM bait) compared to the control for three cherry tree varieties: Regina, Lapins, and Stella. In all varieties, a significantly higher number of flies were captured with the bait compared to the control (*p* ≤ 0.05). Regina captured an average of 16,104 flies/ha with the bait and 1944 flies/ha in the control. Lapins recorded the highest number of flies captured with bait, reaching 22,491 flies/ha, while 8330 flies/ha were recorded in the control. Finally, Stella showed captures of 13,050 flies/ha with bait and 3887 flies/ha in the control. This demonstrates the effectiveness of the bait in attracting flies compared to the control treatment across the three varieties (Table 3).

## 4. Discussion

### 4.1. Olfactometric Bioassays

Previous studies have demonstrated that olfactory signals from damaged and fermented fruits play a crucial role in food source recognition by SWD [52,53]. These flies are typically attracted to fermented sweet substrates, such as decaying fruits. However, their attraction has also been reported to increase in response to the aromas of wines, vinegars, and fermentation volatiles, including acetic acid and ethanol [23,54,55].

For instance, studies employing gas chromatography coupled with electroantennographic detection (GC-EAD), along with laboratory bioassays and field capture experiments, were conducted to identify the volatile compounds from wine and vinegar involved in the attraction of SWD [22,52]. In addition to acetic acid and ethanol, consistent GC-EAD responses were observed for 13 volatile compounds from wine and 7 from vinegar, with all active GC-EAD compounds from vinegar also present in wine. In a field capture experiment, both the 9-component vinegar blend and the 15-component wine blend were equally attractive when compared to a blend of acetic acid and ethanol, but they were less attractive than the combined wine and vinegar blend [52]. The above is consistent with the results obtained in the olfactometric assays presented in this study, where flies exhibited a significant preference for the WVM stimulus (Figure 1), a blend of wine and vinegar. Furthermore, this attraction response is consistent with findings reported by Liu et al. [56], who studied the same dipteran species using olfactometric techniques and volatiles from *Osyris wightiana*, a wild host plant classified as a secondary host of SWD. The authors reported a significant attraction of SWD to the fruits and volatile extracts of *O. wightiana* compared to the control (65–85%), which aligns with the attraction range observed in this study (70%). In all cases, as in our study, SWD were strongly attracted to the stimuli, likely due to the characteristic fermentation aromas [57]. Interestingly, SWD exhibited a “repellent” behavior to SAG I and SAG II baits under laboratory conditions, contrary to what happens under field conditions. A possible explanation for this behavior could be due to the volume used in the olfactometric tests (10 µL) compared to the traps used for monitoring, which contain over 250 mL of bait [48]. Research with mosquitoes (Diptera) refers to the care that must be taken when replicating the concentration of compounds in olfactometric tests to which the insect is accustomed in nature [58]. In addition, SWD’s olfactory receptors may not adapt to these baits in such low volumes or concentrations. On the contrary, SWD may be able to adapt its olfactory receptors to the WVM bait because this bait is also composed of wine, which has a high content of volatile organic compounds, increasing the possibility of receiving them. However, these hypotheses need to be investigated and verified in future work.

### 4.2. Release Rate

Fincheira et al. assessed the thermal properties of nanostructured lipid carriers containing volatile organic compounds as growth inducers in vegetables. This was accomplished by weighing a known sample (20 mg) on a tared ceramic tray and subsequently heating it to measure compound release [45,46]. Their study is complemented by the release kinetics results obtained in this study, which demonstrated the release of compounds from the encapsulated WVM bait in an alginate matrix over a period of 120 h. Furthermore, recent reports by Agnich et al. [59] employed the Korsmeyer–Peppas model to investigate the release pharmacokinetics of nanoemulsions containing *Cymbopogon pendulus* (lemongrass) essential oil, achieving compound viability for 50 days. Similarly, the studies by Fincheira et al. [45,46] demonstrated the integrity of their capsules across different temperature ranges, with stability observed between 20 °C and 30 °C. This is consistent with the results from the controlled environment chamber experiment, where the bait’s durability was evaluated at a temperature of 21.2 °C and a humidity of 75%, maintaining integrity and compound release over a period of 25 days. These findings confirm the success of encapsulation compared to liquid baits. Finally, the use of sodium alginate demonstrated promising results in terms of conservation and durability, in addition to being biocompatible, non-toxic, and biodegradable [60]. The simplicity of capsule manufacturing suggests the potential for large-scale production, with the future exploration of alternative encapsulating materials not excluded.

### 4.3. Field Trial

Traps are essential for pest monitoring, but adult captures do not always accurately reflect insect population densities or predict fruit infestation levels [55]. The earliest traps for SWD consisted of plastic bottles with liquid bait, followed by commercial container-type traps using the same bait [20]. Both designs pose challenges for counting and identification, as flies often degrade in the liquid medium.

For instance, our field evaluation of encapsulated bait confirmed the strong preference of SWD for the WVM bait compared to the control (Figure 5), supporting previous research conducted in Brazil and Italy [40,61]. A total of 78.5% of flies were captured in traps baited with WVM bait, while only 21.5% were caught in control traps. These findings reinforce the effectiveness of this bait for monitoring SWD in cherry orchards. In addition, these results become even more remarkable when the fly population on the plants is extrapolated to one hectare, making the bait’s attraction power much more numerically evident. In terms of sex distribution, 95.3% of the captured flies were females, while only 4.7% were males. This disparity can be attributed to the greater attraction of immature reproductive females to fermented baits rather than ripe fruits, whereas males use traps primarily to locate mates [62]. Monitoring data revealed that October had the highest SWD captures. Additionally, during this month, most varieties at the Maquehue Experimental Field were in bloom, likely enhancing olfactory stimuli that attract SWD. Although temperature is a fundamental factor for the increase in the SWD population in a given time, there are other equally important factors that contribute to this effect such as humidity, photoperiod, available diet, and pressure from predators or competitors [63,64,65].

With respect to the effect of cherry varieties on SWD capture, the Lapins variety showed the highest number of captured individuals, followed by Regina and Stella. These findings align with the study by Buzzetti [66], which reported high SWD infestation rates in Lapins and Regina varieties in the Ñuble Region, leading to significant economic losses. Preliminarily, the use of microencapsulated bait proved effective in preserving the bait’s attractiveness and enhancing SWD capture. This new format, combined with delta-type adhesive traps, offers a valuable tool for generating SWD population curves in southern Chile, which is crucial for informed pest control decision-making [62].

## 5. Conclusions

These results indicate that it is feasible to optimize a system for monitoring and capturing a pest insect such as SWD. The use of a microencapsulated attractant (WVM) in delta-type traps allowed for the capture of a significantly higher number of females compared to males. This is advantageous because capturing females and preventing their mating can reduce infestation levels in ripe fruits, which are preferred by gravid females for oviposition. Additionally, measuring the release rate of the attractant enabled us to estimate its durability under specific weather conditions for a given area. Future research should focus on (1) determining the electrophysiological response of SWD to volatiles from WVM bait, (2) evaluating this monitoring system in commercial cherry orchards, (3) assessing its efficacy in other SWD host plants, and (4) evaluating the capture efficiency of SWD in traps baited with encapsulated WVM versus WVM in liquid format. This information will help to better understand the behavior of SWD in our country, particularly in generating SWD flight curves in areas where it is already established, which is a critical component of an effective integrated pest management (IPM) program for this economically significant dipteran.

## Figures and Tables

**Table 1:** Detailed formulation table for each of the baits used in field trials to attract spotted wing drosophila adults.

Bait	Ingredients
SAG I	Apple cider vinegar: 900 mL Ethyl alcohol 95°: 100 mL
SAG II	Sugar: 2 g Yeast: 0.325 g Wheat flour: 17.25 g Apple cider vinegar: 1 mL Water: 25 mL
WVM	Merlot wine: 600 mL Apple cider vinegar: 400 mL Sugar cane molasses: 20 g

**Table 2:** Agroclimatic parameters for the 2022 season. Agro-meteorological Network INIA, Maquehue Station, Freire, La Araucanía Region.

Date	Temperature (°C)	Minimum Temperature (°C)	Maximum Temperature (°C)	Relative Humidity (%)	Accumulated Precipitation (mm)
Sep-2022	9	2.8	15.2	88.1	44
Oct-2022	11.1	5.2	16.9	83.3	116.5
Nov-2022	15.2	9	21.5	80.1	38.2
Dec-2022	16.6	9.6	23.7	71.9	14

**Table 3:** Number of flies captured per hectare (flies/ha) in traps with WVM bait and in the control for three fruit tree varieties (Regina, Lapins, and Stella). The asterisk (*) indicates significant differences (*p* < 0.05) between the bait treatment and the control within each variety.

Variety	WVM Bait (fly/ha)	Control (fly/ha)
Regina	16,104 *	1944
Lapins	22,491 *	8330
Stella	13,050 *	3887

## Data Availability

The data presented in this study are available upon request from the corresponding author.

## References

[1] Kanzawa T. (1939). Studies on *Drosophila suzukii* Mats. Rev. Appl. Entomol..

[2] Kanzawa T. (1936). Studies on *Drosophila suzukii* Mats. J. Plant Prot..

[3] Dos Santos L.A., Mendes M.F., Krüger A.P., Blauth M.L., Gottschalk M.S., Garcia F.R. (2017). Global potential distribution of *Drosophila suzukii* (Diptera, Drosophilidae). PLoS ONE.

[4] Hauser M. (2011). A historic account of the invasion of *Drosophila suzukii* (Matsumura) (Diptera: Drosophilidae) in the continental United States, with remarks on their identification. Pest Manag. Sci..

[5] SENASICA (2013). Mosca Del Vinagre de Alas Manchadas (Drosophila suzukii Matsumura).

[6] Deprá M., Poppe J.L., Schmitz H.J., De Toni D.C., Valente V.L.S. (2014). The first records of the invasive pest *Drosophila suzukii* in the South American continent. J. Pest Sci..

[7] González G., Mary A.L., Goñi B. (2015). *Drosophila suzukii* (Matsumura) found in Uruguay. Drosoph. Inf. Serv..

[8] Santadino M.V., Riquelme M.B., Ansa M.A., Bruno M., Di Silvestro G., Lunazzi E.G. (2015). Primer registro de *Drosophila suzukii* (Diptera: Drosophilidae) asociado al cultivo de arándanos (*Vaccinium* spp.) de Argentina. Rev. Soc. Entomol. Arg..

[9] Bizama G. (2020). Invasión de *Drosophila suzukii* (Matsumura) en Chile: Utilizando los modelos de distribución de especies como herramienta de bioseguridad. Rev. Chil. Entomol..

[10] European and Mediterranean Plant Protection Organization (EPPO) (2016). *Drosophila suzukii* Does Not Occur in Chile. https://gd.eppo.int/reporting/article-5875.

[11] (2017). SAG Resolución Exenta Nº3672: Medidas Fitosanitarias de Emergencia Provisionales Para la Plaga Drosófila de Alas Manchadas-*Drosophila suzukii* (Matsumura) Diptera: Drosophilidae. División Protección Agrícola y Forestal, Ministerio de Agricultura de Chile/Departamento de Sanidad Vegetal. http://www.diariooficial.interior.gob.cl/publicaciones/2017/06/20/41788/01/1229986.pdf.

[12] Walsh D.B., Bolda M.P., Goodhue R.E., Dreves A.J., Lee J.C., Bruck D.J., Walton V.M., O’Neal S.D., Zalom F.G. (2011). *Drosophila suzukii* (Diptera: Drosophilidae): Invasive pest of ripening soft fruit expanding its geographic range and damage potential. J. Integr. Pest Manag..

[13] Rota-Stabelli O., Blaxter M., Anfora G. (2013). Quick Guide: *Drosophila suzukii*. Curr. Biol..

[14] Bolda M., Goodhue R., Zalom F. (2010). 2010. Spotted wing drosophila: Potential economic impact of a newly established pest. Agric. Resour. Econ..

[15] Yeh D., Drummond F., Gómez M., Fan X. (2020). The Economic Impacts and Management of Spotted Wing Drosophila (*Drosophila suzukii*): The Case of Wild Blueberries in Maine. J. Econ. Entomol..

[16] Thistlewood H., Gill P., Beers E., Shearer P., Walsh D., Rozema B., Acheampong S., Castagnoli S., Yee W., Smytheman P. (2018). Spatial Analysis of Seasonal Dynamics and Overwintering of *Drosophila suzukii* (Diptera: Drosophilidae) in the Okanagan-Columbia Basin, 2010–2014. Environ. Entomol..

[17] Cini A., Ioriatti C., Anfora A. (2012). A review of the invasion of *Drosophila suzukii* in Europe and a draft research agenda for integrated pest management. Bull. Insectol..

[18] Jaffe B.D., Avanesyan A., Bal H.K., Feng Y., Grant J., Grieshop M.J., Lee J.C., Liburd O.E., Rhodes E., Rodriguez-Saona C. (2018). Multistate comparison of attractants and the impact of fruit development stage on trapping *Drosophila suzukii* (Diptera: Drosophilidae) in raspberry and blueberry. Environ. Entomol..

[19] Lee J.C., Bruck D.J., Curry H., Edwards D.L., Haviland D.R., Van Steenwyk R.A., Yorgey B.M. (2011). The susceptibility of small fruits and cherries to the spotted wing drosophila, *Drosophila suzukii*. Pest Manag. Sci..

[20] Lee J.C., Shearer P.W., Barrantes L.D., Beers E.H., Burrack H.J., Dalton D.T., Dreves A.J., Gut L.J., Hamby K.A., Haviland D.R. (2013). Trap Designs for Monitoring *Drosophila suzukii* (Diptera: Drosophilidae). Environ. Entomol..

[21] Lee J.C., Burrack H.J., Barrantes L.D., Beers E.H., Dreves A.J., Hamby K.A., Haviland D.R., Isaacs R., Richardson T.A., Shearer P.W. (2012). Evaluation of monitoring traps for *Drosophila suzukii* (Diptera: Drosophilidae) in North America. J. Econ. Entomol..

[22] Kleiber J.R., Unelius C.R., Lee J.C., Suckling D.M., Qian M.C., Bruck D.J. (2014). Attractiveness of fermentation and related products to spotted wing Drosophila (Diptera: Drosophilidae). Environ. Entomol..

[23] Burrak H.J., Asplen M., Bahder L., Collins J., Drummond F.A., Guédot C., Issacs R., Johnson D., Blanton A., Lee J.C. (2015). Multistate Comparison of Attractants for Monitoring *Drosophila suzukii* (Diptera: Drosophilidae) in Blueberries and Caneberries. Environ. Entomol..

[24] Groupe de Travall Baies (2015). Drosophila suzukii: Strategie 2015 pour les petits fruits. Agroscope Fiche Technique N° 20.

[25] Kirkpatrick D.M., McGhee P.S., Gut L.J., Miller J.R. (2017). Improving monitoring tools for spotted wing drosophila, *Drosophila suzukii*. Entomol. Exp. Appl..

[26] Clymans R., Van Kerckvoorde V., Bangels E., Akkermans W., Alhmedi A., De Clercq P., Beliën T., Bylemans D. (2019). Olfactory preference of *Drosophila suzukii* shifts between fruit and fermentation cues over the season: Effects of physiological status. Insects.

[27] Del Fava E., Ioriatti C., Melegaro A. (2017). Cost–benefit analysis of controlling the spotted wing drosophila (*Drosophila suzukii* (Matsumura)) spread and infestation of soft fruits in Trentino, Northern Italy. Pest Manag. Sci..

[28] Food and Agriculture Organization of the United Nations/World Health Organization (FAO/WHO) (2010). FAO/WHO Expert Meeting on the Application of Nanotechnologies in the Food and Agriculture Sectors: Potential Food Safety Implications.

[29] Mutis A., Parra L., Manosalva L., Palma R., Candia O., Lizama M., Pardo F., Perich F., Quiroz A. (2010). Electroantennographic and behavioral responses of adults of raspberry weevil *Aegorhinus superciliosus* (Coleoptera: Curculionidae) to odors released from conspecific females. Environ. Entomol..

[30] Khot L.R., Sankaran S., Maja J.M., Ehsani R., Schuster E.W. (2012). Applications of nanomaterials in agricultural production and crop protection: A review. Crop Prot..

[31] Ghormade V., Deshpande M.V., Paknikar K.M. (2011). Perspectives for nano-biotechnology enabled protection and nutrition of plants. Biotechnol. Adv..

[32] Brignone S.G., Ravetti S., Palma S.D. (2020). Microencapsulación/recubrimiento de sistemas particulados de uso farmacéutico. Pharm. Technol. Ed. Sudam..

[33] Ríos-Aguirre S., Gil-Garzón M.A. (2021). Microencapsulación por secado por aspersión de compuestos bioactivos en diversas matrices: Una revisión. TecnoLógicas.

[34] De Oliveira J., Ramos Campos E., Bakshi M., Abhilash P.C., Fernandes Fraceto L. (2014). Application of nanotechnology for the encapsulation of botanical insecticides for sustainable agriculture: Prospects and promises. Biotechnol. Adv..

[35] Koschier E., De Kogel W., Visser J. (2000). Assessing the attractiveness of volatile plant compounds to western flower thrips *Frankliniella occidentalis*. J. Chem. Ecol..

[36] Renkema J.M., Buitenhuis R., Hallett R.H. (2014). Optimizing Trap Design and Trapping Protocols for *Drosophila suzukii* (Diptera: Drosophilidae). J. Econ. Entomol..

[37] Lasa R., Tadeo E., Toledo-Hernández R.A., Carmona L., Lima I., Williams T. (2017). Improved capture of *Drosophila suzukii* by a trap baited with two attractants in the same device. PLoS ONE.

[38] Carroll J. (2016). Spotted Wing Drosophila (SWD) Monitoring Traps; Program Based on Methods Tested by Steven Alm, Dept. of Plant Sciences and Entomology, University of Rhode Island, Richard Cowles, Connecticut Agricultural Experiment Station and Greg Loeb, Dept. of Entomology, Cornell University; EEUU. https://www.google.com/url?sa=t&source=web&rct=j&opi=89978449&url=https://cpb-us-e1.wpmucdn.com/blogs.cornell.edu/dist/0/7265/files/2017/01/SWDTraps_CornellFruit-1vwi4oo.pdf&ved=2ahUKEwjfz_-Mqr-KAxUB4jQHHZUnN4EQFnoECBoQAQ&usg=AOvVaw3nE2rk2VV_RkxMwh-_F3O5.

[39] (2017). Manejo Cultural de Drosophila suzukii; SAG Ficha Técnica N°1; Subdepartamento Moscas de la Fruta/Departamento de Sanidad Vegetal/División Protección Agrícola y Forestal/SAG/Ministerio de Agricultura de Chile: Santiago, Chile. https://www.google.com/url?sa=t&source=web&rct=j&opi=89978449&url=https://www.sag.gob.cl/sites/default/files/ficha_drosophila_n1.pdf&ved=2ahUKEwiYi8OhvL-KAxUioK8BHVqFCLMQFnoECB0QAQ&usg=AOvVaw0a46DohqqdHzdJP5Id2iQj.

[40] Wollmann J., Schlesener D.C.H., Vieira J.G.A., Bernardi D., Garcia M.S., Garcia F.R.M. (2019). Evaluation of Food Baits to Capture *Drosophila suzukii* in the Southern of Brazil. An. Acad. Bras. Ciênc..

[41] Espinoza J., Chacón-Fuentes M., Quiroz A., Bardehle L., Escobar-Bahamondes P., Ungerfeld E. (2021). Antifeedant Effects and Repellent Activity of Loline Alkaloids from Endophyte-Infected Tall Fescue against Horn Flies, *Haematobia irritans* (Diptera: Muscidae). Molecules.

[42] Riyajan S.-A., Sakdapipanich J. (2009). Encapsulated neem extract containing Azadiractin-A within hydrolyzed poly(vinyl acetate) for controlling its release and photodegradation stability. Chem. Eng. J..

[43] Hamby K.A., Kwok R.S., Zalom F.G., Chiu J.C. (2013). Integrating circadian activity and gene expression profiles to predict chronotoxicity of *Drosophila suzukii* response to insecticides. PLoS ONE.

[44] Red Agrometeorológica de INIA (2022). Estación Maquehue UFRO, Freire. https://agrometeorologia.cl/.

[45] Fincheira P., Jofré I., Tortella G., Medina C., Quiroz A., Seabra A.B., Nascimento H.M., Diez M.C., Rubilar O. (2021). The Prospection of Plant Response to 2-Ketones Released from Nanostructured Lipid Carriers. J. Soil Sci. Plant Nutr..

[46] Fincheira P., Rubilar O., Tortella G., Medina C., Seabra A.B., Nascimento M., Diez M.C., Quiroz A. (2021). Formulation of a Controlled-Release Carrier for 2-ketones Based on Solid Lipid Nanoparticles to Increase Seedling Growth in *Lactuca sativa* and *Solanum lycopersicum*. J. Soil Sci. Plant Nutr..

[47] Spies J., Liburd O. (2019). Comparison of attractants, insecticides, and mass trapping for managing *Drosophila suzukii* (Diptera: Drosophilidae) in blueberries. Fla. Entomol..

[48] (2017). Detección de Insectos Adultos de *Drosophila suzukii* por Medio del uso de Trampas; SAG Ficha Técnica N°2; Subdepartamento Moscas de la Fruta/Departamento de Sanidad Vegetal/División Protección Agrícola y Forestal/SAG/Ministerio de Agricultura de Chile: Santiago, Chile. https://www.google.com/url?sa=t&source=web&rct=j&opi=89978449&url=https://www.sag.gob.cl/sites/default/files/ficha_drosophila_n2.pdf&ved=2ahUKEwjf3YHKvb-KAxXlhq8BHbVEFp0QFnoECB0QAQ&usg=AOvVaw0JwEQemqpBHedOgv9q0Ma5.

[49] Rojas O., Andrade J., Concha C., Astudillo F. (2019). Estados de Desarrollo de Drosophila suzukii (Diptera: Drosophilidae) y Otras Especies del Género, Comunes en el sur de Chile.

[50] Ilgin P., Ozay H., Ozay O. (2019). A new dual stimuli responsive hydrogel: Modeling approaches for theprediction of drug loading and release profile. Eur. Polym. J..

[51] Pourtalebi L., Ghazali M., Ashrafi H., Azadi A. (2020). 2020. A comparison of models for the analysis of the kinetics of drug release fromPLGA-based nanoparticles. Heliyon.

[52] Cha D.H., Adams T., Rogg H., Landolt P.J. (2012). Identification and Field Evaluation of Fermentation Volatiles from Wine and Vinegar that Mediate Attraction of Spotted Wing Drosophila, *Drosophila suzukii*. J. Chem. Ecol..

[53] Asplen M.K., Anfora G., Biondi A., Choi D.S., Chu D., Daane K.M., Gibert P., Gutierrez A.P., Hoelmer K.A., Hutchison W.D. (2015). Invasion biology of spotted wing Drosophila (*Drosophila suzukii*): A global perspective and future priorities. J. Pest Sci..

[54] Dzialo M.C., Park R., Steensels J., Lievens B., Verstrepen K.J. (2017). Physiology, ecology and industrial applications of aroma formation in yeast. FEMS Microbiol. Rev..

[55] Tait G., Mermer S., Stockton D., Lee J., Avosani S., Abrieux A., Anfora G., Beers E., Biondi A., Burrack H. (2021). *Drosophila suzukii* (Diptera: Drosophilidae): A Decade of Research Towards a Sustainable Integrated Pest Management Program. J. Econ. Entomol..

[56] Liu Y., Cui Z., Shi M., Kenis M., Dong W., Zhang F., Zhang J., Xiao C., Chen L. (2021). Antennal and Behavioral Responses of *Drosophila suzukii* to Volatiles from a Non-Crop Host, *Osyris wightiana*. Insects.

[57] Cloonan K.R., Abraham J., Angeli S., Syed Z., Rodríguez-Saona C. (2018). Advances in the chemical ecology of the spotted wing Drosophila (*Drosophila suzukii*) and its applications. J. Chem. Ecol..

[58] Raji J.I., Melo N., Castillo J.S., González S., Saldana V., Stensmyr M.C., DeGennaro M. (2019). *Aedes aegypti* mosquitoes detect acidic volatiles found in human odor using the IR8a pathway. Curr. Biol..

[59] Agnish S., Sharma A.D., Kaur I. (2022). Nanoemulsions (O/W) containing *Cymbopogon pendulus* essential oil: Development, characterization, stability study, and evaluation of in vitro anti-bacterial, anti-inflammatory, anti-diabetic activities. Bionanoscience.

[60] Funami T., Fang Y., Noda S., Ishihara S., Nakauma M., Draget K., Nishinari K., Phillips G. (2009). Rheological properties of sodium alginate in an aqueous system during gelation in relation to supermolecular structures and Ca^2+^ binding. Food Hydrocoll..

[61] Tonina L., Grassi A., Caruso S., Mori N., Gottardello A., Anfora G., Giomi F., Vaccari G., Ioriatti C. (2018). Comparison of attractants for monitoring *Drosophila suzukii* in sweet cherry orchards in Italy. J. Appl. Entomol..

[62] Swoboda-Bhattarai K.A., McPhie D.R., Burrack H.J. (2017). Reproductive Status of *Drosophila suzukii* (Diptera: Drosophilidae) Females Influences Attraction to Fermentation-Based Baits and Ripe Fruits. J. Econ. Entomol..

[63] Hamby K.A., Bellamy D.E., Chiu J.C., Lee J.C., Walton V.M., Wiman N.G., York R.M., Biondi A. (2016). Biotic and abiotic factors impacting development, behavior, phenology, and reproductive biology of *Drosophila suzukii*. J. Pest Sci..

[64] Wallingford A.K., Lee J.C., Loeb G.M. (2016). The influence of temperature and photoperiod on the reproductive diapause and cold tolerance of spotted-wing Drosophila, *Drosophila suzukii*. Entomol. Exp. Appl..

[65] Guédot C., Avanesyan A., Hietala-Henschell K. (2018). Effect of temperature and humidity on the seasonal phenology of *Drosophila suzukii* (Diptera: Drosophilidae) in Wisconsin. Environ. Entomol..

[66] Buzzetti K. (2020). The Spotted Wing Drosophila in the South of the World: Chilean Case and Its First Productive Impacts. Invasive Species—Introduction Pathways, Economic Impact, and Possible Management Options.

